# 235 Diagnostic Qualities of the Short Form of the Bruininks-Oseretsky Test of Motor Proficiency for Low Motor Competence

**DOI:** 10.1093/eurpub/ckae114.161

**Published:** 2024-09-26

**Authors:** Iva Šeflová, Josef Chudoba

**Affiliations:** Technical University of Liberec, Liberec, Czech Republic; Technical University of Liberec, Liberec, Czech Republic

## Abstract

**Purpose:**

The Bruninks Oseretsky Test of Motor Proficiency, 2nd Edition (BOT 2) diagnostic tool with 53 test tasks is used for objective assessment of motor proficiency with regard to the diagnosis of motor deficits in motor control. The normative criteria are initially validated on the US population ages 4 - 21. An adaptation for the European setting exists for German-speaking countries ages 4 - 14. There are short versions of the BOT 2 with self-selected items for screening purposes.

The aim of our study was to evaluate the diagnostic quality of a German short version of BOT 2 for low motor competence and for the risk of developmental coordination disorder.

**Methods:**

The motor competence level in n = 637 Czech children aged 6.0-11.0 years (46.6% girls) was evaluated using Total motor composite and subcategories of fine and gross motor skills: Fine manual control, Manual coordination, Body coordination and Strength and agility.

We used the following variables to describe the diagnostic validity: Sensitivity, Specificity, Accuracy, Positive and Negative Predictive Values, the Receiver Operating Characteristic, and the Area Under the Curve.

**Results:**

The short version of the BOT 2 shows a sensitivity for low motor competence of 77%, specificity of 86% and validity of 83%. For the risk of developmental coordination disorder, sensitivity is 77%, specificity is 94%, and validity is 93%. However, there is a lower positive predictive value of 49%.

**Conclusions:**

The German short version of BOT 2 is an alternative test to the BOT 2 complete form, which is a suitable diagnostic tool for assessing low motor competence, including the diagnosis of risk for DCD. Overall, the BOT 2 short form provides a lower Total motor composite score than the BOT 2 complete form and places significantly more children in the well-below-average category. This is reflected in the low predictive value of a positive test.

**Funding Source:**

This work was supported by the Technology Agency of the Czech Republic under Grant TA ČR Éta 3 TL03000221.

